# Effect of mild medical hypothermia on in vitro growth of *Plasmodium falciparum* and the activity of anti-malarial drugs

**DOI:** 10.1186/s12936-016-1215-8

**Published:** 2016-03-15

**Authors:** Khalid Rehman, Ulrich Sauerzopf, Luzia Veletzky, Felix Lötsch, Mirjam Groger, Michael Ramharter

**Affiliations:** Division of Infectious Diseases and Tropical Medicine, Department of Medicine I, Medical University of Vienna, Währinger Gürtel 18-20, 1090 Vienna, Austria; Centre de Recherches Médicales de Lambaréné, Hôpital Albert Schweitzer, Lambaréné, Gabon; Institut für Tropenmedizin, Universität Tübingen, Tübingen, Germany

**Keywords:** Malaria, In vitro, HRP2, Hypothermia, Drug activity, *P. falciparum*

## Abstract

**Background:**

Cerebral malaria remains a medical emergency with high mortality. Hypo-perfusion due to obstructed blood vessels in the brain is thought to play a key role in the pathophysiology of cerebral malaria leading to neurological impairment, long-term neuro-cognitive sequelae and, potentially, death. Due to the rapid reversibility of vascular obstruction caused by sequestered *Plasmodium falciparum*, it is hypothesized that mild medical hypothermia—a standard intervention for other medical emergencies—may improve clinical outcome. This preclinical in vitro study was performed to assess the impact of mild hypothermia on parasite growth and the intrinsic activity of anti-malarials drugs.

**Methods:**

Three laboratory-adapted clones and two clinical isolates were used for growth assays and standardized drug sensitivity assessments using the standard HRP2 assay. All assays were performed in parallel under normothermic (37 °C), mild hypothermic (32 °C), and hyperthermic (41 °C) conditions.

**Results:**

Parasite growth was higher under standard temperature condition than under hypo- or hyperthermia (growth ratio 0.85; IQR 0.25–1.06 and 0.09; IQR 0.05–0.32, respectively). Chloroquine and mefloquine had comparable in vitro activity under mild hypothermic conditions (ratios for IC50 at 37 °C/32 °C: 0.88; 95 % CI 0.25–1.50 and 0.86; 95 % CI 0.36–1.36, respectively) whereas dihydroartemisinin was more active under mild hypothermic conditions (ratio for IC50 at 37 °C/32 °C: 0.27; 95 % CI 0.19–0.27). Hyperthermia led by itself to almost complete growth inhibition and precluded further testing of the activity of anti-malarial drugs.

**Conclusion:**

This preclinical evaluation demonstrates that mild medical hypothermia inhibits in vitro growth of *P. falciparum* and enhances the pharmacodynamic activity of artemisinin derivatives. Based on these preclinical pharmacodynamic data, the further clinical development of mild medical hypothermia as adjunctive treatment to parenteral artesunate for cerebral malaria is warranted.

## Background

Cerebral malaria is a medical emergency requiring the immediate parenteral administration of effective anti-malarial treatment in addition to best available intensive care. Cerebral malaria leads to high mortality and neurological and cognitive long-term sequelae even in settings where optimal intensive care is available [1]. The underlying pathophysiological mechanism is thought to be facilitated by the adherence of erythrocytes infected with *Plasmodium falciparum* trophozoites to the endothelial cell wall of small cerebral blood vessels with consecutive hypo-perfusion of the brain. This hypo-perfusion, which may lead to progressively increased intracranial pressure, is, however, rapidly reversible by the administration of anti-malarial drugs [2]. Clearance of parasites from peripheral blood is usually achieved within 24 h in most African settings, where the burden of severe malaria is highest—constituting a relatively short period of time for vulnerability of brain tissue [3].

Mild medical hypothermia is an established adjunctive treatment initiated immediately after successful resuscitation from sudden cardiac arrest. It has been demonstrated to improve neurological outcome in cardiac arrest survivors due to its ability to increase the permissive period of hypoperfusion of the brain and to attenuate reperfusion injury [4, 5]. A target temperature of 32 °C is recommended for cardiac arrest patients and mild medical hypothermia is currently evaluated for other indications including ischemic stroke [4, 6].

Based on the analogy of the reversibility of hypoperfusion of the brain in cardiac arrest and in cerebral malaria, it is hypothesized that mild medical hypothermia has the potential to improve neurological and cognitive outcomes of patients treated for cerebral malaria. However, before testing this hypothesis in human patients, the preclinical evaluation of the effect of hypothermia on the growth of *P. falciparum* and the potential impact on the activity of anti-malarial drugs require investigation. Although hypothermia has been previously shown to reduce growth of *P. falciparum* [7, 8], this has not been investigated in the target range of mild medical hypothermia. Importantly, the effect of mild medical hypothermia on the pharmacodynamics of anti-malarial drugs has not been investigated before. This is particularly important as hypothermia may—at least in theory—lead to reduced anti-malarial activity due to reduced metabolic activity of the parasite and may thus put patients participating in such clinical trials at potential risk [9]. The aim of this study was, therefore, to evaluate the impact of hypothermia on the in vitro growth of *P. falciparum* and on the in vitro activity of anti-malarial drugs.

## Methods

Five *P. falciparum* strains were used for this in vitro study, including three clones with distinct anti-malarial resistance patterns (7G8, DD2, 3D7) and two laboratory-adapted isolates from returning travellers from West Africa. Clone 7G8 is resistant to chloroquine and pyrimethamine. Clone DD2 shows resistance to pyrimethamine, mefloquine and chloroquine. Clone 3D7 is drug sensitive [10]. The laboratory-adapted strains were previously shown to exhibit high-level drug resistance against chloroquine but are sensitive to mefloquine and artemisinin derivatives. Parasites were kept in continuous malaria culture until optimum growth was achieved prior to growth inhibition assays as described previously [11, 12].

Growth inhibition assays were performed using a modified HRP2-assay protocol [13, 14]. All growth assays were performed in parallel under identical conditions except for incubation temperature. Temperatures were set at 37, 41 and 32 °C using calibrated incubators. Culture plates were pre-dosed with ascending concentrations of the anti-malarial drugs chloroquine diphosphate (Sigma C6628), mefloquine and dihydroartemisinin. Stock solutions (1 mg/ml) of drugs were prepared in ethanol and further diluted to the required final concentrations (chloroquine 3200 nmol/l, mefloquine 160 nmol/l and dihydroartemisinin 16 nmol/l). Two-fold serial dilutions were employed and drug-free controls were performed in parallel.

Two-hundred microliter parasite culture with a parasitaemia of 0.01 % and haematocrit of 1.5 % were transferred to each of the drug-coated wells. All assays were performed in triplicate and negative controls were immediately frozen at initiation of the incubation period.

Growth was assessed by detection of HRP2 concentrations by ELISA using commercially available monoclonal antibodies (1 µg/ml of IgM antibody MPFM-55 and IgG MPFG-55P55A, Immunology Consultants Laboratory, Portland, OR, USA). Inhibitory concentrations (IC) were calculated using freely available software (IVART) [15]. To obtain relative growth rates, logistic growth curves were fitted to data points obtained by serial dilution of lysed cultures using a least square regression model. Logistic regression analysis was conducted using the “R” statistical package [16]. No ethical clearance was required for this in vitro study following regulations of the Ethics Committee of the Medical University of Vienna.

## Results

Laboratory-adapted clones (n = 3) and clinical isolates (n = 2) were cultured in vitro until optimal growth was achieved. All parasites were then subjected in parallel to growth and growth inhibition assays under normothermic (37 °C), hyperthermia (41 °C) and mild hypothermic (32 °C) conditions. All strains showed adequate growth under normothermic conditions as assessed by the increase of HRP2 concentrations in parasite culture (median increase 3.9; IQR 3–7.8). Hypothermia was associated with a markedly reduced growth rate compared to normothermia (relative growth rate 0.85; IQR 0.25–1.06). Hyperthermia led to the highest inhibition of parasite growth (0.09; IQR 0.05–0.32); see (Table 1).

**Table 1:** In vitro growth of *Plasmodium falciparum* and inhibitory concentrations of dihydroartemisinin chloroquine, and mefloquine under normo-, hypo- and hyperthermic conditions

Ratio of growth rate	32 °C	IQR	37 °C	41 °C	IQR	
Isolate 1	1.15		1	0.16		
Isolate 2	0.97		1	0.48		
DD2	0.22		1	0.04		
3D7	0.85		1	0.07		
7G8	0.28		1	0.09		
Total	0.85	0.25–1.06		0.09	0.05–0.32	

	50 % inhibitory concentration of anti-malarial drugs (nmol/l and IQR)					
	CQ 32 °C	CQ 37 °C	DHA 32 °C	DHA 37 °C	MQ 32 °C	MQ 37 °C
Isolate 1	75.0 (40.2–109.8)	141.3 (124.9–157.6)	0.36 (0.30–0.43)	1.14 (0.96–1.32)	11.9 (10.2–13.6)	14.3 (11.7–17.0)
Isolate 2	69.2 (55.7–82.8)	153.8 (130.1–177.5)	0.24 (0.17–0.31)	1.38 (1.19–1.57)	6.8 (5.1–8.6)	17.7 (15.9–19.4)
DD2	337.4 (235.0–439.8)	167.6 (139.3–195.9)	0.49 (0.36–0.63)	1.23 (1.05–1.41)	26.3 (17.0–35.6)	14.7 (11.3–18.1)
3D7	38.6 (26.8–50.5)	33.9 (23.1–44.6)	0.44 (0.32–0.55)	1.51 (1.37–1.65)	26.6 (19.9–33.3)	30.0 (27.0–33.0)
7G8	46.7 (25.8–67.5)	190.8 (178.4–203.1)	0.20 (0.16–0.24)	1.12 (1.03–1.20)	2.2 (1.9–2.4)	5.4 (4.4–6.4)

*IQR* interquartile range, *95* *% CI* 95 % confidence intervals, *CQ* chloroquine, *DHA* dihydroartemisinin, *MQ* mefloquine

The anti-parasitic activity of the anti-malarial drugs chloroquine, dihydroartemisinin and mefloquine was assessed under the three respective temperature conditions (Table 1). Chloroquine and mefloquine had comparable IC50 values at 32 and 37 °C (chloroquine: 113 nmol/l; 95 % CI 2.8–224 nmol/l vs 137 nmol/l; 95 % CI 84–191 nmol/l and mefloquine 15 nmol/l; 95 % CI 5–25 nmol/l and 16 nmol/l; 95 % CI 9–24 nmol/l, respectively). However, dihydroartemisinin showed considerably lower IC50 values under mild hypothermic conditions (0.3 nmol/l; 95 % CI 0.2–0.5 nmol/l vs 1.3; 95 % CI 1.1–1.4 nmol/l; p < 0.01).

## Discussion

This study demonstrates that mild hypothermia leads in itself to a considerable inhibition of parasite growth. This pharmacodynamic activity is independent of the action of anti-malarials and is therefore most likely unaffected by the presence of specific drug resistance. Interestingly, hyperthermia is associated with an even higher inhibition of in vitro parasite growth. However, hyperthermia is likely to increase the metabolic activity of brain tissue and therefore potentially leads to adverse outcome of cerebral malaria. Based on these data, the current clinical practice of normalizing temperature mechanically or with the use of antipyretic drugs may ultimately prove deleterious based on the finding of maximum in vitro parasite growth under normothermic temperature conditions. While the inhibitory effect of hyperthermia and hypothermia has been described previously, this is the first systematic evaluation of mild medical hypothermia and its inhibitory effect on *P. falciparum* in the context of anti-malarial drugs. These preclinical findings are further corroborated by clinical observations of faster parasite clearance in human patients in the absence of antipyretic drugs [17, 18].

It is well established that the early administration of parenteral artesunate is the most important treatment of severe malaria [1]. In vitro data from this study indicate that the anti-malarial activity of artesunate and its active metabolite dihydroartemisinin may be enhanced in two ways by mild hypothermia—firstly by direct inhibition of parasite growth and secondly by an increased anti-malarial activity of dihydroartemisinin when used under hypothermic conditions. Based on these findings there is—at least from a pharmacodynamic perspective—no convincing evidence precluding the use of mild medical hypothermia in the treatment of cerebral malaria patients. Whereas this study was performed with the concept of applying mild medical hypothermia in cerebral malaria, this pharmacodynamic activities may also apply to other forms of severe malaria. Similarly, the findings of retained or even increased anti-malarial activity as demonstrated for mefloquine, chloroquine and dihydroartemisinin in this in vitro study may apply to other classes of anti-malarials.

This study is a preclinical pilot study and is not designed to address all potential issues in the context of the use of medical hypothermia in malaria patients. Particularly the effect of mild medical hypothermia on cytoadherence can be investigated further in preclinical studies. Importantly, immunological consequences of hypothermia cannot be fully addressed by such in vitro experiments and the pharmacokinetics of anti-malarial drugs may potentially be altered by the use of mild medical hypothermia. These important aspects should therefore be diligently assessed in the context of the clinical development of hypothermia as adjunct treatment of cerebral malaria. Finally, the sample size of this pilot study is limited and the in vivo parasite reduction rate will therefore be an important outcome of future clinical trials [19].

The further development of mild therapeutic hypothermia may well continue directly in clinical trials in humans given the low predictability of animal models for cerebral malaria [20]. Whereas the conduction of such pilot studies in high-resource settings may be a viable option, endemic regions may be more relevant given the overall burden of disease. Importantly, external mechanical cooling of patients is feasible with relatively simple devices, including cooling blankets, but adequate monitoring of core temperature by oesophageal or urinary bladder probes is mandatory to avoid iatrogenic risks associated with hypothermia below 32 °C [4].

## Conclusion

In summary, this in vitro study provides evidence supporting the clinical development of mild medical hypothermia for the treatment of cerebral malaria. Future studies should include feasibility outcomes of the procedure as well as pharmacodynamic and pharmacokinetic assessments associated with the use of mild medical hypothermia.
